# Partial Decellularization for Segmental Tracheal Scaffold Tissue Engineering: A Preliminary Study in Rabbits

**DOI:** 10.3390/biom11060866

**Published:** 2021-06-10

**Authors:** Luong Huu Dang, Yuan Tseng, How Tseng, Shih-Han Hung

**Affiliations:** 1 International Ph.D. Program in Medicine, College of Medicine, Taipei Medical University, Taipei 110, Taiwan; luonghuudang167@gmail.com; 2 Department of Otolaryngology, Faculty of Medicine, University of Medicine and Pharmacy at Ho Chi Minh City, Ho Chi Minh City 70000, Vietnam; 3 Graduate Institute of Medical Sciences, College of Medicine, Taipei Medical University, Taipei 110, Taiwan; m120109010@tmu.edu.tw; 4 Department of Biochemistry and Molecular Cell Biology, School of Medicine, College of Medicine, Taipei Medical University, Taipei 110, Taiwan; 5 Department of Otolaryngology, School of Medicine, College of Medicine, Taipei Medical University, Taipei 110, Taiwan; 6 Department of Otolaryngology, Wan Fang Hospital, Taipei Medical University, Taipei 116, Taiwan

**Keywords:** partial decellularization, tissue engineering, tracheal transplantation

## Abstract

In this study, we developed a new procedure for the rapid partial decellularization of the harvested trachea. Partial decellularization was performed using a combination of detergent and sonication to completely remove the epithelial layers outside of the cartilage ring. The post-decellularized tracheal segments were assessed with vital staining, which showed that the core cartilage cells remarkably remained intact while the cells outside of the cartilage were no longer viable. The ability of the decellularized tracheal segments to evade immune rejection was evaluated through heterotopic implantation of the segments into the chest muscle of rabbits without any immunosuppressive therapy, which demonstrated no evidence of severe rejection or tissue necrosis under H&E staining, as well as the mechanical stability under stress-pressure testing. Finally, orthotopic transplantation of partially decellularized trachea with no immunosuppression treatment resulted in 2 months of survival in two rabbits and one long-term survival (2 years) in one rabbit. Through evaluations of posttransplantation histology and endoscopy, we confirmed that our partial decellularization method could be a potential method of producing low-immunogenic cartilage scaffolds with viable, functional core cartilage cells that can achieve long-term survival after in vivo transplantation.

## 1. Introduction

Tracheal damage typically results from trauma, neoplastic diseases, congenital anomalies, and intubation procedures. Severe injury or damage to the structure or luminal surface of the trachea can also significantly reduce patient quality of life due to difficulties with breathing, speaking, and swallowing [1]. Anastomoses can be performed to reconnect two tracheal segments after resection for tracheal lesions less than 5 cm in length. However, for defects larger than 5 cm, there are few therapeutic options, which represents a major challenge in trachea reconstruction [2]. Current tracheal reconstructions predominantly involve autologous tissue transplantation, such as a local pedicle flap, or allografts from different donor tissues, such as aortic allograft transplantation [3]. Several clinical and experimental studies have shown that homograft tracheal transplantation is one of the most promising methods for treating major tracheal lesions. However, very few studies regarding long-term successful transplantation in humans have been reported [4]. So far, an ideal solution for tracheal replacement has not been found. This encourages scientists to increased effort in this research field.

In recent decades, there has been a large amount of research focusing on the application of tissue engineering technique in tracheal replacement to overcome the dissatisfaction with conventional therapies [5]. The fabrication of a more applicable tracheal scaffold is complicated as it involves the integration of different components, including the respiratory epithelium for the regeneration of a lining layer, and cartilage components to provide structural support. Recently, a combination of biodegradable scaffolds using 3D printing and cell seeding with bioreactor for fabricating a graftable trachea appeared as a promising method [6,7,8]. However, these reconstruction approaches have been focused on maintaining the integrity of the tubular structure, and problems such as foreign body reactions, difficulty of revascularization, and complexity of ex-vivo cell expansion have not been fully addressed, with the long-term therapeutic effects of this strategy remaining uncertain.

Altenatively, other approaches using decellularization techniques have been proposed and developed for tracheal replacements with varying degrees of success. Decellularization of donor tracheal grafts using various methods, including detergent and enzyme treatment [9,10,11], high hydrostatic pressure technique [12,13], freeze-drying and sonication treatment [14], or a complex combination method [15,16] have been reported and received the most attention. Depite some encouraging results from studies applying the complete decellularization techniques on tracheal reconstructions of a patch tracheoplasty model, the effectiveness of decellularization of the whole segment of the tracheal tube was limited due to scaffold degradation and the lack of new cartilage formation.

While the freeze-dry-sonication-sodium dodecyl sulfate (FDSS) decellularization method efficiently removed the cellular content of the tracheal scaffold in our previous study [14], the process of cartilage cell removal caused significant damage, resulting in the tracheal scaffold being unable to withstand the lumen pressure and the subsequent collapse of the tissue transplant, causing the death of the experimental animals.

The “viability” of the tracheal scaffold to be transplanted seems to be of critical importance. Recellularization with stem cells is to be avoided due to complexity considerations [17,18]; however, preserving some of the cartilage cells during the decellularization process might be an alternative option, as cartilage tissue is thought to have low immunogenicity [19]. More recently, some tissue engineers have shifted their attention toward biological scaffolds fabricated by partial decellularization processing. Preliminary results have been reported in some in-vitro studies; however, the procedure needs to be validated in orthotopic transplantations using end-to-end anastomosis of the recipient trachea with the transplanted scaffold [20,21]. Based on the aforementioned issues, we hypothesized that it would be possible to transplant the scaffold without a severe rejection reaction occurring, even if some of the donated cartilage cells are still alive. Additionally, retaining a portion of viable, functional cartilage cells in the transplant process was expected to contribute to the structural strength of the tracheal scaffold after transplantation, which is critical for maintaining the tracheal tube structure.

In this study, we evaluated a newly developed procedure for the rapid partial decellularization of harvested trachea, producing low-immunogenic cartilage scaffolds with viable, functional core cartilage cells, which can achieve long-term survival after in vivo orthotopic transplantation.

## 2. Materials and Methods

### 2.1. Animals and Study Design

**Figure 1:**
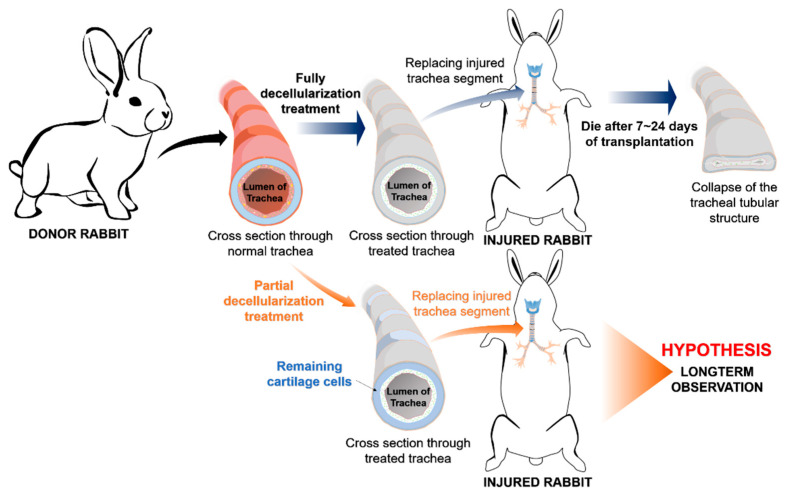
Illustration of the novel concept of the partial decellularization method applied in this study.

The animal use protocol listed below has been reviewed and approved by the Institutional Animal Care and Use Committee (Approval No. LAC-2016-0363). Figure 1 shows the general concept of this preliminary study. All experiments in this study were performed in triplicate for reproducibility and consistency. A total of twelve 9-month-old male New Zealand White rabbits, acquired from BioLASCO company (Taipei, Taiwan) with a bodyweight ranging from 3 kg to 3.5 kg, were subjected to this study. Animals were housed individually in our Experimental Animal Center under standard conditions (temperature 22–24 °C; 12 h light/12 h dark cycle, and free access to food and water) during the study. Specific monitoring criteria will be notified below according to each experiment. The anesthesia process was initiated with the combination of 0.1 mL/kg Zoletil100 and 0.4 mL/kg Rompun intramuscularly. All efforts were made to minimize suffering throughout the experimental process. Rabbits were euthanized either at the end of experiments or when a humane endpoint was reached, whichever came first. Humane endpoints for all experiments were defined as 10% acute weight loss or clinical signs consistent with severe dyspnea, altered mentation and/or anorexia. Euthanasia was performed by using CO_2_ gas after inducing general anesthesia with Zoletil 100 intramuscularly.

### 2.2. Manufacturing the Trachea Scaffold

#### 2.2.1. Partial Decellularization of the Rabbit Trachea

In this study, we used a novel decellularization method (Figure 1), the modified partial decellularization (PD) method, compared to our previous study [14]. This modified decomposition protocol involved a rapid sonication method with two buffers, SDS and PBS. There were six harvested tracheas from six rabbits, which were separated randomly for use in heterotopic implantation and orthotopic transplantation with 3 samples for each group. Specifically, the segment below the cricoid level was harvested and trimmed into 1-cm-long segments. Each tissue sample was subjected to the ultrasonic decellularization process in both buffers as follows: 1 min sonication with 1% SDS followed by 1 min sonication with 1% PBS, repeated 3 times (3 cycles). The power of the sonicator (Elma S 60 H) was set at 180 W. All of the tracheal scaffold segments were processed with 3 full cycles of the PD process and stored in an antibiotic solution.

#### 2.2.2. Decellularized Scaffold Evaluation—Vital Staining Test

To demonstrate that the partial decellularization method is capable of eliminating all mucosal cells and retaining cartilage cells in rabbit trachea, the vital staining method was performed. Briefly, the samples were washed with PBS and submerged in Dulbecco’s modified Eagle’s medium (DMEM; Gibco, Taipei, Taiwan). The cartilage samples were adhered with glue to a small metal block, and 30 µm thick sections were cut using vibratome at a slow speed and medium amplitude. The sections were incubated with 60 mM propidium iodide and 10 mM fluorescein diacetate in PBS in the dark for 5 min. Sections were then washed with PBS for 1 min to remove the residual dye from the tissue matrix. The stained samples were observed under fluorescence microscopy.

### 2.3. Heterotopic Implantation of the Decellularized Graft

#### 2.3.1. Surgical Procedure

**Figure 2:**
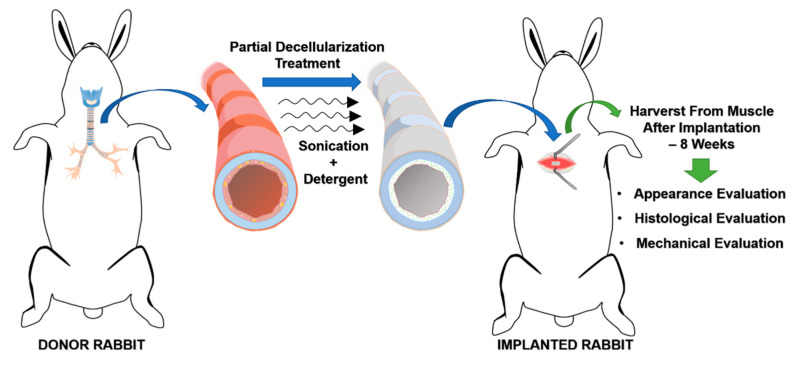
Illustration of heterotopic implantation of the partially decellularized graft into the chest muscle of rabbits.

After the partial decellularization process and the determination of the viability of the cartilage cells, the tracheal allograft segments were circumferentially implanted into the chest muscles of three different rabbits, as illustrated in Figure 2. No immunosuppressive drugs were given to the animals during this process. After implantation, general conditions, vital signs, and body weight were recorded. Analgesics (acetaminophen 100 mg/kg/day) was supplied in the daily drinking water for three consecutive days. Eight weeks after implantation, the animals were sacrificed for histopathological evaluation to reveal the degree of integration of the tracheal scaffold implants.

#### 2.3.2. Histological Evaluations and Mechanical Testing for Heterotopic Transplants

The samples were fixed for 24 h in a 10% neutral buffered formalin solution in PBS (pH 7.4) at room temperature. The samples were washed with distilled water, dehydrated in graded alcohol, embedded in paraffin (Merck, Darmstadt, Germany), and sectioned at 5 µm. The adjacent sections were stained with hematoxylin and eosin (H&E) (Sigma, St. Louis, MO, USA) and observed under a microscope (BA400; Motic, Xiamen, China).

In the mechanical test section, 1 cm rabbit tracheal segments were used. After preparation, the tracheal segments underwent mechanical testing using a universal testing machine (LF Plus, Lloyd Materials Testing, Bognor Regis, UK). The tubes were placed with the long axis in a horizontal position in the compression mode, and the tracheal segments were then compressed laterally. The stress-strain curves of the partially decellularized tracheal segments were recorded and compared with those of native rabbit tracheas to evaluate the collapse-resisting ability of the partially decellularized tracheal scaffolds.

### 2.4. Allograft Orthotopic Transplantation

#### 2.4.1. Surgical Procedure

The orthotopic transplantation was performed on three other healthy rabbits. After the administration of anesthesia, a longitudinal surgical incision was made at the anterior midline of the neck from the hyoid bone level to the lower neck. After dissection through the skin and subcutaneous tissue, the strap muscles were retracted bilaterally, and the trachea was exposed. A 1 cm segment of the trachea was transected and removed, leaving a segment defect in the trachea. The partially decellularized tracheal scaffold was then secured into this defect with an end-to-end anastomosis using 3-0 Vicryl^®^ bioabsorbable sutures. After transplantation, the animal was kept in the animal room and orally administered with antibiotics (enrofloxacin 10 mg/kg/day), analgesics (acetaminophen 100 mg/kg/day), and mucolytic medications (acetylcysteine 100 mg/kg/day) for two weeks. During this time, the animals were strictly observed for any sign of progressively severe dyspnea or any reached humane endpoint.

#### 2.4.2. Endoscopic and Histological Analyses after Allograft Transplantation

Endoscopic observation of the trachea was performed under standard anesthesia in all rabbits 2 months after transplantation using the Olympus distal chip flexible endoscope system (ENF-V2; Olympus, Tokyo, Japan). Endoscopy was performed to examine the condition of the airway lumen of the implanted part of the trachea. The investigated conditions included the status of airway narrowing (stenosis), the color and morphology of the implanted portion relative to the native tissue, and whether there was evidence of tissue destruction.

After the endoscopic examinations, two host-rabbits that exhibited signs of severe difficulty breathing were euthanized. The remaining transplanted rabbit showed mild signs of difficulty breathing, and thus was not sacrificed and maintained for observation for up to 2 years.

For all three rabbits, the post-mortem tracheas were harvested with the transplanted segment for histological analysis. The samples were longitudinally cut, then fixed for 24 h in a 10% neutral buffered formalin solution in PBS (pH 7.4) at room temperature, washed with distilled water, dehydrated in graded alcohol, embedded in paraffin (Merck, Darmstadt, Germany), and sectioned at 5 µm. Adjacent sections were stained with hematoxylin and eosin (H&E) (Sigma, St. Louis, MO, USA) and observed under a microscope (BA400; Motic, Xiamen, China).

## 3. Results

### 3.1. Partially Decellularized Trachea Scaffold Manufacturing and Evaluation

**Figure 3:**
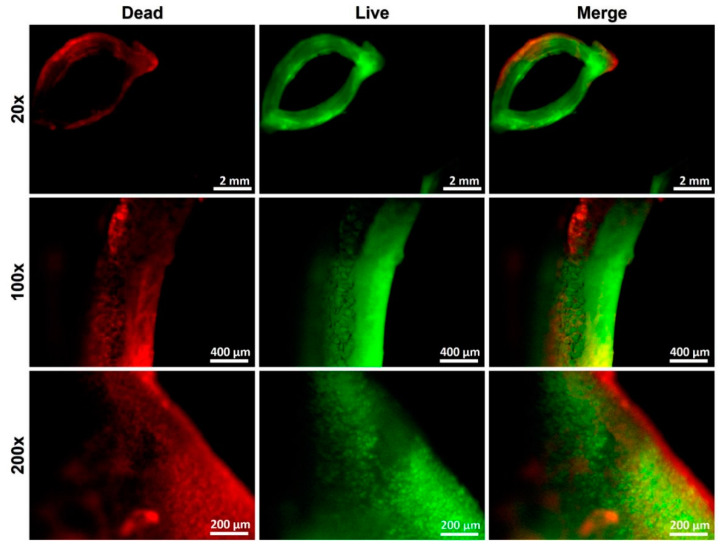
Vital staining of the tracheal segment immediately after the partial decellularization process. Red represents cells that have been damaged by the decellularization process, and green represents cells that are still viable. After the partial decellularization process, the cells outside the cartilage were no longer viable, but the core cells were preserved and remained potentially functional.

After partial decellularization processing, the viability of the remaining chondrocytes in the scaffold could be clearly observed through vital staining, as shown in Figure 3, where red represents cells that have been damaged by the decellularization process, and green represents viable cells. Through the merged images, it can be observed that after the partial decellularization process, while the cells outside of the cartilage were no longer viable, the core cells were preserved and potentially remained functional.

### 3.2. Heterotopic Implantation Test of the Allograft

**Figure 4:**
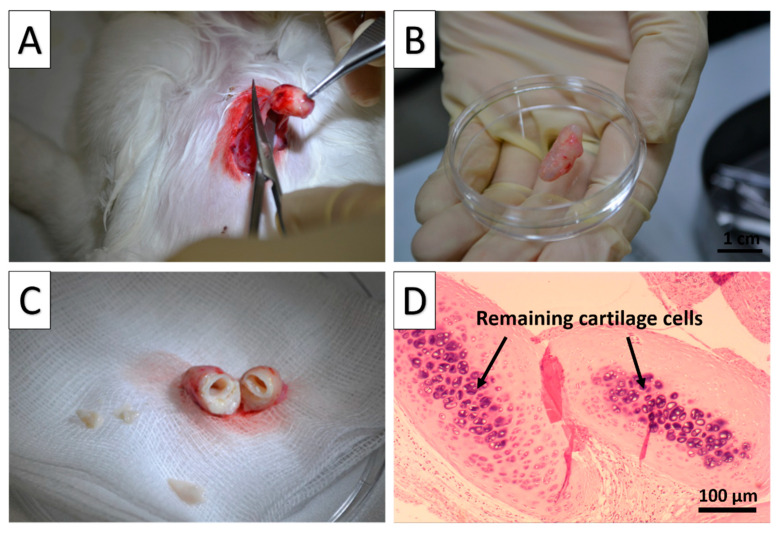
Evaluation of the decellularized tracheal segments after heterotopic muscle implantation. (**A**): Harvest from chest muscle; (**B**): Gross appearance of the harvested trachea 2 months postimplantation; (**C**): Lumen of implanted scaffold appear to be grossly intact; (**D**): H&E staining of harvested trachea 2 months after implantation showed partial cartilage cell loss at the outer layer of the cartilage, while the core remaining cartilage cells were clearly visible (black arrows).

The partial decellularization scaffold was further evaluated with heterotopic implantations into the chest muscle of three rabbits. The tracheal segment was harvested and analyzed after the 8 week implantation period. (Figure 4A).

Grossly, the implanted segments appeared to be intact, elastic, and resistant to tubular collapse of implantation into the rabbits’ chest muscle, and the segments were not filled with other cell types (Figure 4B,C).

In the implanted portion of tissue, no evidence of rejection or tissue necrosis, as a result of autologous immune responses, was observed under no immunosuppressive drugs used during and after the implant process. Under H&E staining, it was observed that the implanted, partially decellularized cartilage showed partial cartilage cell loss at the outer layer of the cartilage. However, the core inner component appeared to be relatively unaffected, with the remaining cartilage cells clearly visible (black arrow), and chondroid was also present in the core section, which was stained more heavily by H&E (Figure 4D).

**Figure 5:**
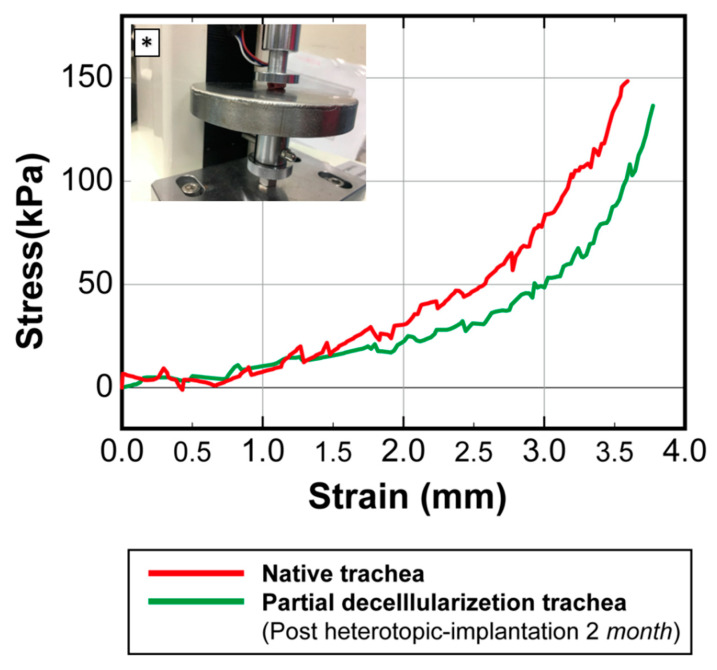
Mechanical strength of the partially decellularized tracheas after 2 months of heterotopic muscle implantation. Compared to that of the native trachea, the curve of the partially decellularized trachea (green curve) showed an approximated similarity, and the mechanical strength was expected to be further restored along with cartilage cell growth. * Mechanical tests under lateral compression settings using a universal testing machine (LF Plus, Lloyd Materials Testing, UK).

The stability of the cartilage framework was also evaluated by a mechanical testing process (Figure 5). The compression curve after heterotopic implantation (green curve) showed that the partially decellularized trachea considerably maintained its durability, with similar mechanical properties compared to that of the native trachea (red curve). In particular, the results in Figure 5 underline the importance of residual cartilage cells to the structural integrity of the tracheal allograft segments and for the enhancement of the segments’ lumen pressure resistance.

### 3.3. Transplantation of the Partially Decellularized Trachea Segment

**Figure 6:**
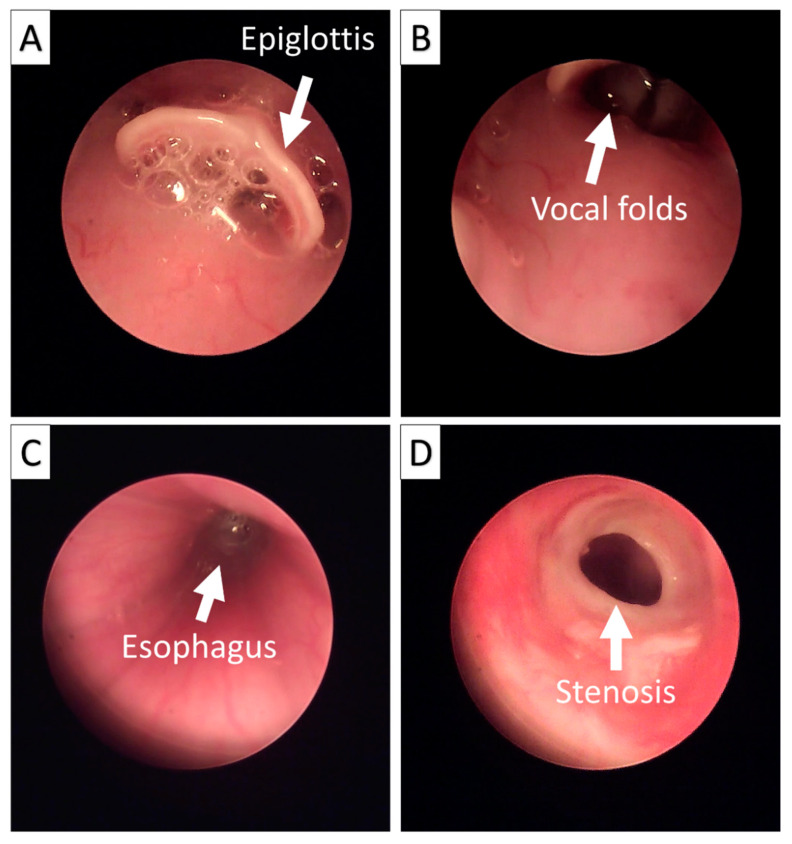
Endoscopic examination of the tracheal allograft segment in rabbits 2 months after orthotopic transplantation. (**A**): Epiglottis; (**B**): Vocal folds; (**C**): Esophagus; (**D**): Circular stenosis ring at the anastomosis site.

In this study, we also performed three different orthotopic transplantations in New Zealand white rabbits. No rabbits died prior to the sacrifice process, demonstrating that the retention of a portion of cartilage in the tracheal cartilage structure through partial decellularization can enhance the ability of the scaffold to withstand the lumen and airway pressure. We also performed an endoscopy on all three of the transplanted rabbits (Figure 6). Phenomena such as stenosis, tissue destruction due to immune rejection, and the airway state of the transplanted segment were investigated. We observed that all three rabbits showed stenosis, but there was no sign of tissue destruction due to immune rejection, despite the fact that we did not provide any immunosuppressant drugs in this study.

However, two out of the three transplanted rabbits showed severe signs of shortness of breath after 2 months of transplantation; thus, we sacrificed these animals and performed H&E staining to examine the transplanted tissue.

**Figure 7:**
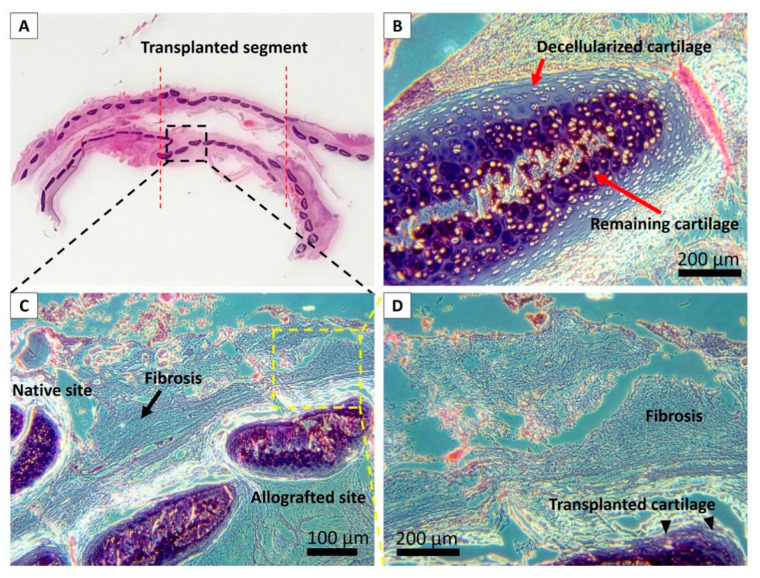
Histological evaluation of the tracheal allograft segment in rabbits two months posttransplantation. (**A**): Microscopic observation of explanted trachea; (**B**): Remained cartilage in the transplanted scaffold; (**C**,**D**): Fibrosis was significantly found at the luminal surface of the transplanted graft, which was accompanied by the incomplete coverage of the regenerated epithelium.

We observed good integration of the partially decellularized scaffold into the host structure under the microscopes with H&E staining, as shown in Figure 7. A portion of cartilage cells remained intact in the partially decellularized scaffold, and we observed very limited histological signs of immuno rejections such as cell infiltration, vascular hyperplasia, tissue necrosis, without applying any immunosuppressive drugs. Fibrosis formation was significantly found, particularly at the middle of the transplanted graft, which was accompanied by the incomplete coverage of the regenerated epithelium.

**Figure 8:**
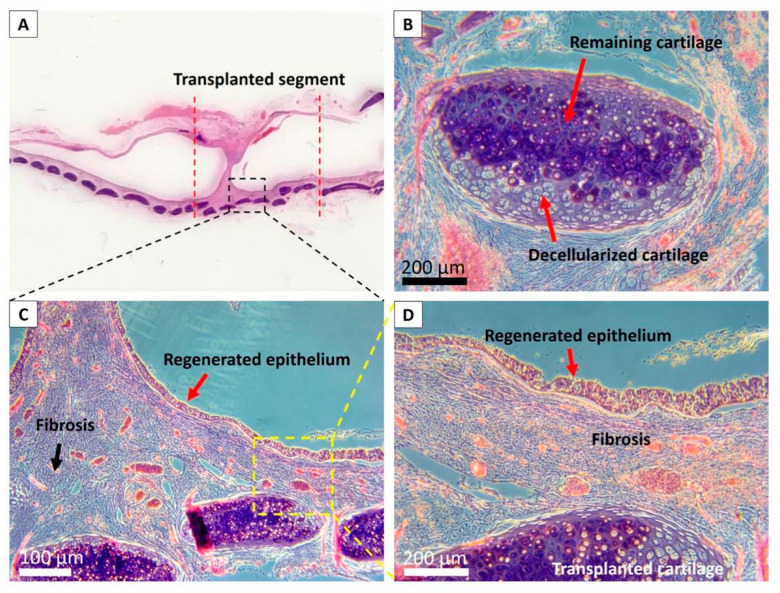
Histological evaluation of the tracheal allograft segment in the rabbit two years posttransplantation. (**A**): Microscopic observation of explanted trachea two years posttransplantation; (**B**): Remained cartilage in the transplanted scaffold; (**C**): The fibrosis had a tendency to develop at the central luminal aspect of the transplanted graft and protruded into the tracheal lumen, forming a circular stenosis ring; (**D**): Complete regenerated epithelium can be observed at the non-stenotic luminal areas.

Despite a long-term follow-up until two years was achieved in one rabbit, non-lethal stenosis at the middle part of the transplanted segment can still be observed. This fibrosis had a tendency to develop at the luminal aspect of the transplanted graft and protruded into the tracheal lumen, forming a circular stenosis ring, which is compatible with the endoscopic findings (Figure 8).

## 4. Discussion

In this study, we demonstrated that through our new partial decellularization protocol, we were able to create tracheal allograft scaffolds that were transplantable in a rabbit model. Through evaluations of post-decellularization structural strength, post-decellularization cell viability, postimplant histology, postimplantation mechanical strength, and posttransplantation histology and endoscopy, we determined that the partially decellularized trachea scaffold procedure appeared to have a potential application for segmental trachea transplantation with the maintenance of long-term tubular integrity of all of the allograft transplanted animals, with no signs of immune rejection.

Based on our previous study, we found that the cartilage cells remained critical and played a role in maintaining airway structure and respiratory capacity after tracheal scaffold transplantation in humans and other mammals [14]. Herein, we designed a new tracheal transplantation method with a quick partial decellularization process by combining detergent and sonication treatment. Partial decellularized tracheal scaffolds were created by using a shorter decellularization time and a reduced number of decellularization cycles than those used in our previous method of decellularization protocol [14], and it was confirmed with a vital assay that the outer epithelial cells were completely removed by the partial decellularization process, while a portion of the core cell of the tracheal cartilage remained viable. Compared to the efficiency of a handful of de-epithelialization techniques that have been previously reported [9,15,22,23], our partial decellularization is more efficient and time-saving, as it is performed in a cocktail style using a combination of sodium dodecyl sulfate—one of the most extensively used detergents for tissue decellularization [20,24,25,26]—and sonication, which greatly improves the efficiency of the decellularization without extensively damaging the desired cartilage cells.

We expected the scaffold to perform differently than in our previous study as the cartilage cells were successfully preserved by the new protocol. As reported in our previous study, the fully decellularized segments were significantly damaged and exhibited degenerative changes 1 week after muscle implantation. Through our new protocol, the transplants appeared to be healthier with an intact lumen. The mechanical compression test demonstrated that the scaffold had the ability to retain the airway structure in the internal body environment during a 2-month period, which our previous freeze-dry-sonication-sodium dodecyl sulfate method failed to achieve, as the tracheal cartilage eventually collapsed. These significant differences strongly indicate that the remaining cartilage cells in the scaffold were not only viable immediately after the decellularization process but also remained functional after transplantation, which is crucial for sustaining the supporting strength of the tracheal tubular structure. The scaffolds demonstrated no signs of necrosis or severe immune rejection under H&E staining. The scaffolds were tested in orthotopic trachea-to-trachea transplantations. As expected, the posttransplantation endoscopic and histological results demonstrated that there was neither a collapse of the transplanted scaffolds, nor severe immune-rejection responses in the recipient animals.

The results of this study show that the values of the partial decellularization process are as follows: (1) The fast efficiency greatly reduces the time needed for scaffold preparation and is more clinically applicable than other methods; (2) the scaffold is prepared with low immunogenic features, and no immunosuppressant use is needed for the recipient animals; (3) the scaffold is decellularized without the disruption of the scaffold architecture, which is important for surgical transplantation procedures and helps the recipient overcome the negative pressure of airflow in the lumen immediately after transplantation; and (4) the viable cartilage cells remained functional after transplantation and gradually restored the mechanical strength similar to that of the native trachea over time.

While the new protocol presented in this study seems to overcome the critical problem of cartilage support of tracheal scaffolds, the transplanted scaffolds eventually failed to serve its functional purpose. There are still some problems that need to be further addressed. In general, there are significant technical requirements and demand for intensive care immediately after transplantation, in which the transplanted cartilage cells are at their most vulnerable stage. Most importantly, mild to moderate stenosis can develop at the luminal aspect of the transplanted graft. In this study, stenosis can be observed 2 months after scaffold transplantation, and remained unresolved for at least 2 years in our longest observations. Additionally, the histologically observed overgrowth of fibroblasts, an undesirable result of primary wound healing [27], at the luminal surface of the transplanted scaffold was significant and protruded into the tracheal lumen, forming a circular stenosis ring.

According to our previous study, the respiratory epithelium seems to heal and migrate rapidly on the surface of the decellularized scaffold. However, the migration of the respiratory epithelium is reported to vary significantly, and cell proliferation and cell migration during respiratory epithelial wound repair differs depending on the cell location within the repair area [28]. The slow migration of epithelial cells might also be influenced by the decellularization process, which completely removes all tissues and blood vessels, which are important factors in promoting epithelial growth [29,30]. These results suggested that the respiratory epithelium component might be the next critical component following the supporting cartilage in the development of an ideal transplantable tracheal scaffold.

In our study, the absence of epithelium was considered an important factor in the induction of fibrosis formation and luminal stenosis, which eventually led to the reduced quality of our tracheal grafts and even death in in vivo animal models. This problem will be intensively investigated in our future work. Possible solutions include cellular therapies [6,31], coating biomedical materials with fibroblast overgrowth inhibitors [32], and applying respiratory epithelium cell sheets for luminal coverage [33,34].

Other limitations of this study should also be noted. First, due to the limitation of sample size, we must be very cautious when analyzing the results, to prevent the risk of over-interpretation. Although the use of the rabbit model has been widely accepted as a standard for research regarding tracheal transplantation [35], there are many issues that need to be considered before applying this technique to humans due to the inevitable inter-species differences. Secondly, the endoscopic and histological results showed that the transplanted partially decellularized tracheal scaffold was low-immunogenic, with no evidence of necrosis and little lymphocyte infiltration. However, these results should be interpreted carefully as the observation of clinical symptoms/signs is limited in animal models, and the mere fact that no immune cells appeared to be present in the histological images does not mean that there was not a long-term immunological response or sustained expression of inflammatory molecules. More specific analysis such as immunohistochemistry staining should be performed. Finally, some limitations in the research methods of this preliminary study whichmade the outcome evaluations inconclusive, including a lack of positive controls in the vital staining assay, a lack of measurement of elastic modulus of transplanted grafts after explantation, and a lack of control group with full decellularization, should be addressed, and need to be improved in our following publications in the near future.

## 5. Conclusions

In conclusion, our fast partial decellularization method was able to create transplantable tracheal allograft scaffolds with preserved viable cartilage cellular components that remain functional, and gradually restore the luminal strength of the tracheal transplant without signs of severe immune rejection, which was critical for the long-term survival of the transplanted animals. Despite the fact that our partially decellularised scaffold eventually did not achieve its functional purpose in this study, it would merit further investigation.
